# The Potential of Cold Atmospheric Pressure Plasmas for the Direct Degradation of Organic Pollutants Derived from the Food Production Industry

**DOI:** 10.3390/molecules29122910

**Published:** 2024-06-19

**Authors:** Piotr Cyganowski, Dominik Terefinko, Agata Motyka-Pomagruk, Weronika Babinska-Wensierska, Mujahid Ameen Khan, Tymoteusz Klis, Wojciech Sledz, Ewa Lojkowska, Piotr Jamroz, Pawel Pohl, Magda Caban, Monica Magureanu, Anna Dzimitrowicz

**Affiliations:** 1 Department of Polymer and Carbonaceous Materials, Wroclaw University of Science and Technology, 27 Wybrzeze St. Wyspianskiego, 50-370 Wroclaw, Poland; 2 Department of Analytical Chemistry and Chemical Metallurgy, Wroclaw University of Science and Technology, 27 Wybrzeze St. Wyspianskiego, 50-370 Wroclaw, Poland; dominik.terefinko@pwr.edu.pl (D.T.); mujahid.khan@pwr.edu.pl (M.A.K.); tymoteusz.klis@pwr.edu.pl (T.K.); piotr.jamroz@pwr.edu.pl (P.J.); pawel.pohl@pwr.edu.pl (P.P.); 3 Laboratory of Plant Protection and Biotechnology, Intercollegiate Faculty of Biotechnology University of Gdansk and Medical University of Gdansk, University of Gdansk, 58 Abrahama, 80-307 Gdansk, Poland; agata.motyka-pomagruk@ug.edu.pl (A.M.-P.); wojciech.sledz@biotech.ug.edu.pl (W.S.); ewa.lojkowska@biotech.ug.edu.pl (E.L.); 4 Research and Development Laboratory, Intercollegiate Faculty of Biotechnology University of Gdansk and Medical University of Gdansk, University of Gdansk, 20 Podwale Przedmiejskie, 80-824 Gdansk, Poland; weronika.babinska-wensierska@ug.edu.pl; 5 Laboratory of Physical Biochemistry, Intercollegiate Faculty of Biotechnology University of Gdansk and Medical University of Gdansk, University of Gdansk, 58 Abrahama, 80-307 Gdansk, Poland; 6 Department of Environmental Analysis, Faculty of Chemistry, University of Gdansk, 63 Wita Stwosza, 80-308 Gdansk, Poland; magda.caban@ug.edu.pl; 7 National Institute for Lasers, Plasma and Radiation Physics, Department of Plasma Physics and, Nuclear Fusion, 409 Atomistilor Str., 077125 Magurele, Romania; monimag@infim.ro

**Keywords:** non-thermal plasma, reactive oxygen and nitrogen species, catalysts, biological effects, multidrug resistance, environmental impact, pollutants removal

## Abstract

Specialized chemicals are used for intensifying food production, including boosting meat and crop yields. Among the applied formulations, antibiotics and pesticides pose a severe threat to the natural balance of the ecosystem, as they either contribute to the development of multidrug resistance among pathogens or exhibit ecotoxic and mutagenic actions of a persistent character. Recently, cold atmospheric pressure plasmas (CAPPs) have emerged as promising technologies for degradation of these organic pollutants. CAPP-based technologies show eco-friendliness and potency for the removal of organic pollutants of diverse chemical formulas and different modes of action. For this reason, various types of CAPP-based systems are presented in this review and assessed in terms of their constructions, types of discharges, operating parameters, and efficiencies in the degradation of antibiotics and persistent organic pollutants. Additionally, the key role of reactive oxygen and nitrogen species (RONS) is highlighted. Moreover, optimization of the CAPP operating parameters seems crucial to effectively remove contaminants. Finally, the CAPP-related paths and technologies are further considered in terms of biological and environmental effects associated with the treatments, including changes in antibacterial properties and toxicity of the exposed solutions, as well as the potential of the CAPP-based strategies for limiting the spread of multidrug resistance.

## 1. Introduction

Among the detrimental effects of human action involving climate change, overusing natural resources, the fragmentation and degradation of natural habitats, decreases in biodiversity, and the introduction of invasive species, contamination of waterways attracts particular interest. Even though water covers 71% of the Earth’s surface, only 3% is freshwater, from which only 0.5% is available for human use [1]. This supply, which is constantly undergoing the hydrologic water cycle, forms a limited reservoir of 8.4 million litres for each person on the globe [1]. Sadly, the naturally occurring purification processes supported by the operation of wastewater treatment plants show highly restrained potency in the removal of biologically active contaminants, including antibiotics and persistent organic pollutants. Therefore, alternative, green approaches need to be implemented for tackling the presence of these residuals in the ecosystem, with special emphasis on water reservoirs. Here, we summarize and discuss current advances in the research aiming to implement cold atmospheric pressure plasmas in the degradation of antibiotics and persistent organics pollutants of anthropogenic origin.

### 1.1. Antimicrobial Agents and Persistent Organic Pollutants 

One of the most significant and ground-breaking discoveries in the history of medicine was the pioneering work of Alexander Fleming in 1928, regarding the disclosure of the first antibiotic: penicillin [2]. Despite the fact that a long time has passed since antibiotic therapies were first implemented into the healthcare system, the production and consumption of these pharmaceuticals is still increasing [3,4,5]. One of the greatest demands for antibiotic production originates from the animal husbandry sector, in which these substances are utilized not only for disease-curing purposes, but also for preventive applications and growth acceleration in the livestock [6,7,8]. Unfortunately, such an overuse of pharmaceuticals leads to frequent occurrence of these drugs or their partially metabolized derivatives in animal urine and/or excrement, in addition to the final food products. Subsequently, the active molecules of antibiotics or the corresponding metabolites permeate into the natural environment, including surface waters, soil, plants, animals, and even humans. In particular, these environmental hazards tend to be accumulated, posing a threat of acute or chronic toxicity following exposure. Adverse effects caused by contact with these residuals are suggested to take place in microorganisms, aquatic wildlife, plants, animals, and humans [6,7,8].

Moving to assessment of the impact of antibiotic residuals on plant physiology, these substances appear to accumulate in roots, which is negative to the overall plant growth, impedes the uptake of water and minerals, and leads to a decrease in dry mass and disturbances in the photosynthesis process [9]. In more detail, during photosynthesis, many disruptions in electron flux are reported to affect the oxidation–reduction potential necessary for energy production. Some studies have revealed that the accumulation of pharmaceuticals is directly involved in plant wilting [6].

Infiltration of the unmetabolized antibiotics into groundwater, and subsequently into larger water reservoirs, represents a great threat to aquatic organisms. The presence of pharmaceuticals in the aquatic environment can directly endanger algae and cyanobacteria, which are crucial for oxygen production and form the foundations of food chains [10,11,12]. Cyanobacteria as prokaryotes are particularly sensitive to the presence of antibiotics [12,13]. Moreover, these pharmaceuticals might directly affect the metabolism of chloroplasts in green algae and, as a result, impede the photosynthetic efficacy. For instance, the toxicity of ciprofloxacin (CFX), belonging to the fluoroquinolone class of antibiotics, measured as a half-maximal effective concentration (EC_50_), was reported to reach 0.005 mg L^−1^ for *Microcystis aeruginosa* (Cyanobacteria) and 1.1 mg L^−1^ for *Pseudokirchneriella subcapicata* (green algae) [14]. Notably, the harmful effects of antibiotics depend not only on the class of antibiotics and studied organisms, but are also associated with their dose and exposure time. For instance, amoxicillin (AMX), belonging to β-lactams, showed EC_50_ values as high as 1000 mg L^−1^ in relation to green algae, which classifies this antibiotic as non-toxic for aquatic microorganisms [14]. On the other hand, green algae exhibited great sensitivity towards ofloxacin (OFX) and CFX, suggesting the interdependence between chemical structure and toxicity of drugs towards aquatic microorganisms [14]. Other factors also impact the susceptibility of aquatic wildlife to common antibiotics; for instance, interdependence between pH and the growth inhibition of *P. subcapitata* after treatment with sulfamethoxazole (SAX) was reported. Some other research disclosed that the algal antioxidant system was notably affected by erythromycin (ERM) and SAX. Interestingly, *P. subcapitata* showed the least tolerance to ERM as compared to the other investigated pharmaceuticals [15]. The aquatic toxicity of antibiotics against invertebrates, in this case *Hydra attenuata, Artemia salina*, *Daphnia magna*, and *Ceriodaphnia dubia*, turned out to be significant as the defined EC_50_ values for ERM, SAX, or oxytetracycline (OTC) were low, and did not reach 1 mg L^−1^ [14]. A harsh response to these pharmaceuticals, particularly present in large concentrations and after their accumulation, was also reported in several fish-oriented studies [16,17,18]. To exemplify, even though toxicity assays on zebrafish (*Danio rerio*) embryos revealed that the lethal concentration that causes death in 50% of test animals used during a toxicity test study (LC_50_) for tetracycline was 500 mg L^−1^, suggesting low acute toxicity, the long-lasting application of 0.1 mg L^−1^ tetracycline resulted in bending and deformation of the embryo spines. Prolonged exposure of these larvae to 0.02 mg L^−1^ tetracycline led to the delayed absorption of yolk sacs and the recognition of uninflected swimming bladders [19]. To conclude, even low concentrations of antibiotics have direct adverse effects on aquatic life [20]. Therefore, there is an urgent need to solve this problem.

**Figure 1:**
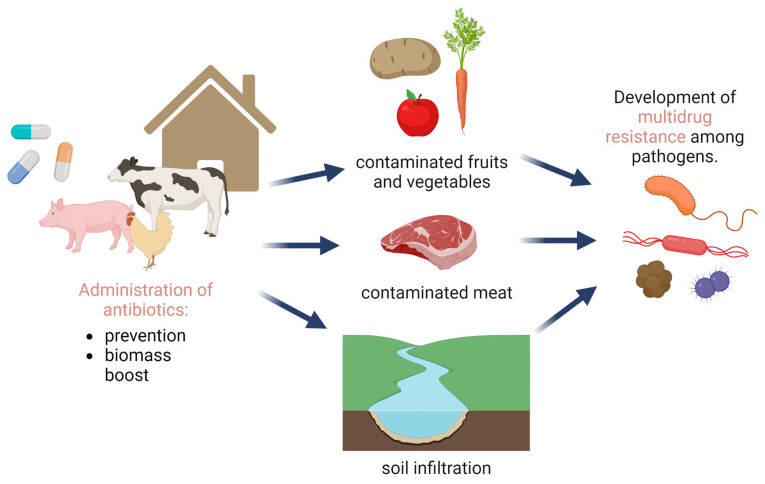
Contribution of antibiotic use in animal husbandry to the spread of multidrug resistance (MDR) among pathogenic microorganisms.

The large-scale production of meat is related to an increase in the density of animal housing. This, in turn, facilitates the spread of epizootics, and thus leads to increased mortality and a drop in the meat production rate [21]. In this context, the use of antibiotics has become a global preventive strategy for boosting financial profits in this sector [22]. This controversial approach has triggered discussion on animal rights and draws attention to the problem of multidrug resistance (MDR) among pathogenic microorganisms [23,24,25,26,27,28]. The scale of this problem finds its reflection in data on the antibiotics use. Currently, 64% of all antimicrobial agents sold are consumed by livestock [29]. As a result, most farm animals carry antibiotic-resistant bacteria [30]. These microorganisms can make contact with humans through the handling of raw meat. Furthermore, residuals of antibiotics or their metabolites may enter the natural environment via different routes. For instance, they can intentionally or unintentionally enter the soil together with manure. Subsequently, antimicrobial agents can be taken up by vegetables that are then often consumed uncooked and/or unprepared [31]. All of these factors (Figure 1) lead to an inevitable risk of MDR development by human pathogens [25,26]. As such, all challenges related to that problem are recognized as a contemporary and “hot topic” perspective to be managed [32].

Although the literature presents technologies such as biotic and abiotic processes (supported by microorganisms or chemical reactions) aiming to effectively decompose hazardous organic compounds from wastewaters [33,34], these approaches, if undertaken alone, may leave a variety of partially decomposed residuals [34,35,36,37]. These substances, in turn, resist different environmental processes [33,38,39], revealing a persistent character. Other organic compounds are used in agroindustry, such as pesticides, in addition to persistent organic pollutants (POPs). The POP group includes various polychlorinated biphenyls (PCBs), organochlorine compounds such as dichlorodiphenyltrichloroethane (DDT), polychlorinated dibenzo-p-dioxins (PCDDs), polychlorinated dibenzofurans (PCDFs), and polychlorinated naphthalenes (data based on the Stockholm Convention) [40,41,42]. These chemicals are easily accumulated in the biosphere, and are identified as extremely harmful to all ecosystem components because they provide substantial damage to regeneration processes in soil and threaten the health of organisms inhabiting various niches. Moreover, most of the negative effects of POPs, such as their long half-lives, the ability to migrate thousands of miles from the source of contamination, and bioaccumulation in the food chain, have not previously been taken into consideration [42,43]. Consequently, the occurrence of POPs attracted the attention of various environmental agencies, including the US Environmental Protection Agency, UN Environmental Programme, and World Health Organisation [44,45,46]. One of the actions undertaken to control POP-related hazards involves the adoption of the Stockholm Convention [43], which resulted in banning the registration of almost all POP-based products. Importantly, DDT is still used for prevention of malaria [47]; thus, due to its unintentional dispersion, long shelf life, and tendency to penetrate into landfill reservoirs, it poses a notable threat that needs to be managed [42,48].

Necessity to address the issues related to antibiotics and POPs is of utmost importance. The extensive use of antibiotics in animal husbandry contributes to the propagation of antimicrobial resistance, which is a global public health concern [20]. Furthermore, antimicrobial agents and POPs tend to accumulate in the biosphere and cause damage to ecosystems, with long-lasting effects on the health and reproduction of organisms, posing various risks to them across different environmental compartments [42,43]. Given the magnitude of these issues and their potential consequences, it is crucial to focus research efforts on the development of effective strategies for the neutralization of these main pollutants from the food production chain. Mitigation of all environmental impacts of antibiotics and POPs is a key aspect in relation to the sustainability of food production systems.

### 1.2. Established Methods for Organic Pollutant Removal

**Figure 2:**
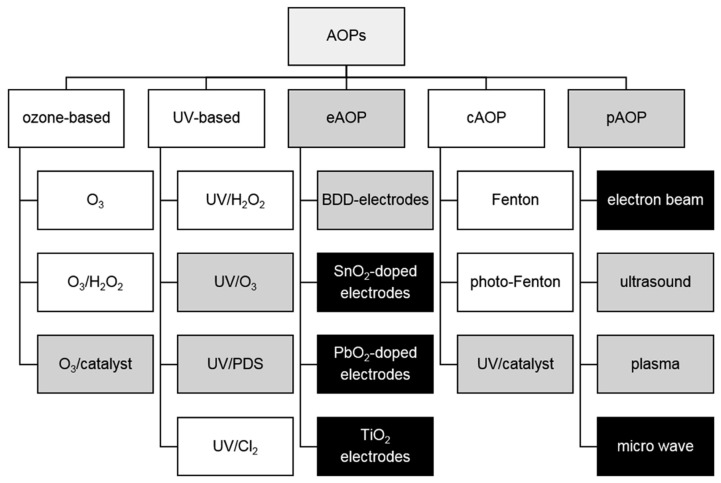
Classification of advanced oxidation processes (AOPs). Different shades mark processes launched on the industrial scale (white), the verified lab- and pilot-scale (grey), and the tested lab-scale (black). Figure reproduced from reference [62] with the permission of Elsevier.

It is well known that antibiotics and organic pollutants exhibit a persistent nature [23,42,43,48]. These chemicals or their intermediates do not easily decompose in natural processes, and cannot be effectively removed by classical wastewater purification methods, including traditional physical or biological processes exploited in wastewater treatment plants [23,49]. These types of contaminants require the implementation of more advanced and alternative approaches. In this context, a series of physiochemical methods, including advanced oxidation processes (AOPs) such as Fenton oxidation processes (FOPs) [50,51,52,53,54,55,56], chlorination [56], ozonation [57,58], and various photo- and catalytical reactions [59,60,61], were proven successful for organic pollutant removal. Among those methods, antibiotics are usually decomposed using a series of AOPs, which are extensively described in the scientific literature [62]. Figure 2 shows diverse AOP-based approaches, implemented in the laboratory as well as on the industrial scale, to decompose such hazardous compounds.

All these methods might be recognized as effective for the decomposition of persistent hazardous compounds. Despite the widespread use of ozonation-based processes, classified to AOPs, there is still a great need for the development of new, effective, and cheap technologies to degrade organic compounds from wastewater. An alternative perspective involves the use of advanced methods such as the decomposition of organic pollutants under microwave radiation in an oxidizing environment [63] or the implementation of ionizing radiation [64]. Nevertheless, additional processing steps (i.e., the adjustment of pH in addition to oxidizing or reducing conditions) are frequently required [63,64].

In this context, cold atmospheric pressure plasma (CAPP) technologies seem to be particularly emerging and interesting. With the use of CAPP-based approaches, there is no need to use additional reagents, while the whole processes can often be carried out under ambient conditions. Considering the above-listed advantages, this literature review provides information on the effectiveness, applications, and development of CAPP-based technologies in terms of neutralizing important organic pollutants such as antibiotics and POPs.

The present review focuses on the main aspects of CAPP-based approaches, enabling a critical assessment of this emerging technology. In this study, we present a summary of the applied CAPP-based strategies, used for removing antibiotics and other selected organic compounds such as pyrene, dichlorvos, melathion, endosulfan, and phenol, with attributing attention to constructions of the systems, their operating parameters, the reached efficiencies, in addition to the achieved deviations in biological activities of the treated pollutants. Moreover, we have shown the function of reactive oxygen and nitrogen species (RONS) for the removal of antibiotics and POPs. Additionally, possible measures for future development are outlined, and critical assessment of the environmental impact is provided. Finally, the future perspectives of CAPP applications are discussed.

## 2. CAPPs for Degrading Organic Pollutants

Plasma is often called the fourth state of matter, arising from the ionization of selected gases by their collision with highly energetic electrons. Among different types of plasma, particular attention should be attributed to CAPP, due to its non-equilibrium conditions and the effective production of RONS such as ^•^OH, ^•^O, H_2_O_2_, NO_x_, and ^•^HO_2_, which are formed during its operation [65].

**Figure 3:**
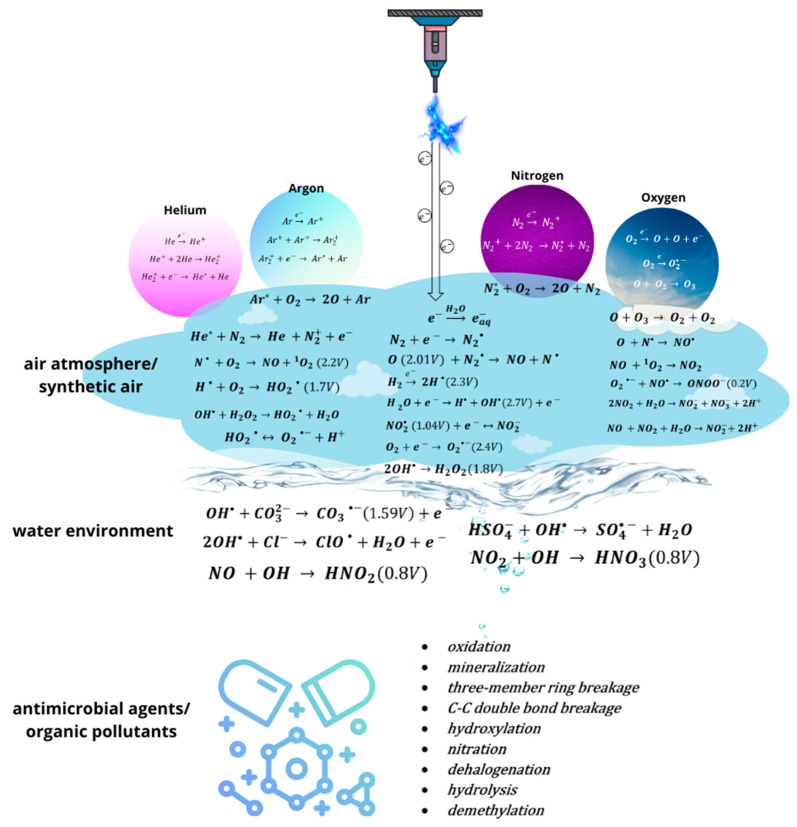
Illustration of possible reactions leading to RONS formation from CAPP discharge applied to pollutants in a liquid environment. In the brackets, the oxidation potential of crucial RONS representatives is mentioned according to the literature [66].

To provide insight into RONS formation, a schematic map illustrating plasma–atmosphere, plasma–liquid and plasma–pollutant interactions, along with the oxidation potential of crucial RONS representatives, is presented in Figure 3. The CAPP is generated in helium, argon, air, nitrogen, or oxygen atmospheres, due to the significant flux of electrons, resulting in the occurrence of metastable atoms. These metastable atoms further interact with surrounding atmosphere constituents, leading to the generation of further RONS representatives. An air atmosphere, as a rich source of nitrogen and oxygen, provides an environment for ROS and RNS generation due to their interactions with metastable atoms and electrons [66]. The free radicals, atomic particles, and ions generated by interacting with each other lead to the generation or recombination of further RONS with certain oxidation activities and lifetimes. The CAPP discharge propagating into the surrounding air finally meets the water environment with dissolved antimicrobial agents and organic pollutants. From this moment, the competing processes occur. RONS with the highest oxidation potential participate in the degradation processes of pollutants, leading to oxidation, mineralization, and a broad spectrum of breakage or the addition/elimination of certain functional groups or atoms in the pollutant’s chemical structure. Finally, the RONS representatives that are excessively produced in CAPP and that do not participate in degradation processes generate the RONS with a longer lifetime [66].

**Figure 4:**
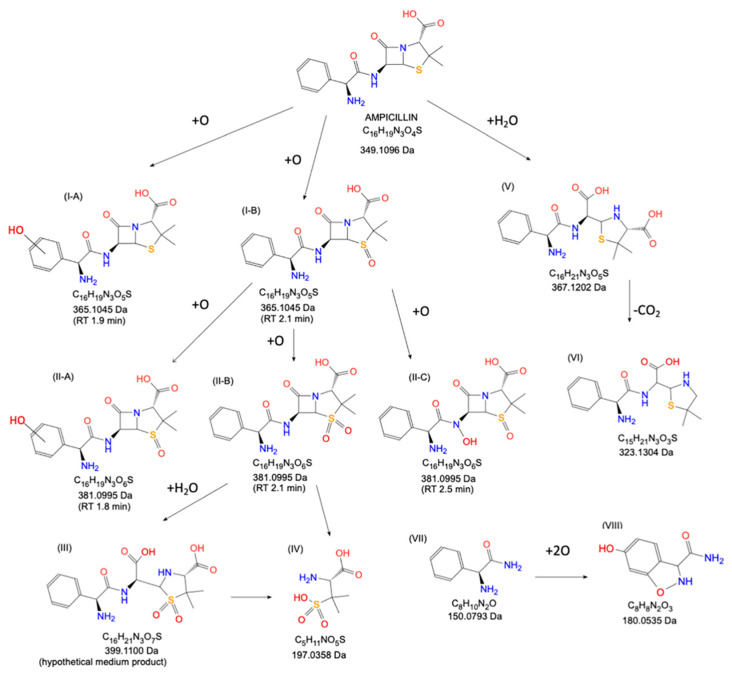
The anticipated CAPP-driven degradation pathways of ampicillin antimicrobial agent. The figure was reproduced from the reference [67] with the permission of Elsevier.

In addition to RONS, solvated electrons, UV radiation, thermal energy, and electromagnetic fields are generated during CAPP operation. Mainly, the above-listed components are responsible for the specific properties of CAPPs, leading to cascade processes responsible for the decomposition of organic compounds, and resulting in the degradation or inactivation of various hazards in diverse liquid or solid matrices [65]. Figure 4 displays an example of the CAPP-driven degradation pathways observed in the processing of ampicillin in simulated wastewater. Such degradation was found to be multi-directional, but the mineralization to low-mass inorganics is the final effect when process is completed.

As a result, CAPPs are widely studied and find plentiful applications in almost every field of knowledge and industry. They are proven to be useful in the medicine and food industries, in addition to environmental protection, because of their implementation in purification processes in gases, waters, and soil, among others [68,69,70,71]. In relation to the latter use, CAPPs might facilitate elimination or reduction in pollutant concentrations via the occurrence of CAPP–liquid interactions [68,69,70], which involve the generation of different reactive species during CAPP operation. The presence of RONS promotes the CAPP-generated environment, facilitating the decomposition of various pollutants without the addition of any external agents that could be considered as a contaminant at the end of the process [72,73]. In this context, CAPP-based technology might be recognized as an eco-friendly, direct approach that enables the effective decomposition of organic pollutants without considerable side effects. Considering the novelty of the CAPP-based methods, these emerging technologies have gained considerable attention in recent years, which is documented in a few review papers summarizing the mechanisms and different settings of CAPP operations [68,69,70]. As the decomposition of various hazardous compounds by CAPPs does not require any additional reagents to be applied, this method is recognized as eco-friendly. In addition, CAPP is responsible for high decomposition yields and efficient degradation rates [74,75]. For this reason, a number of reviews published in recent years focuses on the CAPP-mediated deactivation of organic pollutants [71,73,75,76,77,78,79,80,81]. However, compared to previous review papers [71,73,75,76,77,78,79,80,81], this article undertaes not only the topic of water purification from antibiotics [71,73,75,76,77,78,79,80,81] and POPs [75], but also, discusses in detail the applied plasma sources and drugs degradation mechanisms, in addition to the observed changes in antimicrobial properties, and toxicity of the CAPP-treated organic compounds.

### 2.1. CAPP-Based Technologies for the Removal of Antimicrobial Agents and Organic Pollutants from Different Matrices

**Figure 5:**
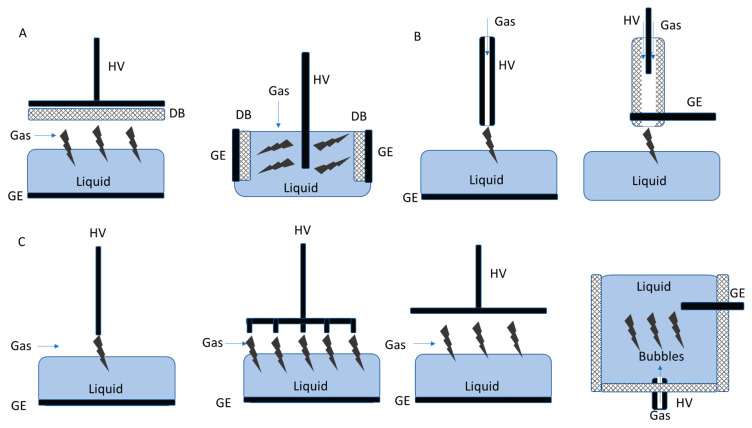
The configurations of CAPP reactors applied for the wastewater purification process. The labels are as follows: (**A**) DBD reactors; (**B**) APPJ reactors; and (**C**) corona or glow discharge reactors. Abbreviations used in the graphic: HV, high-voltage electrode; GE, grounded electrode; and DB, dielectric barrier.

Aiming for the efficient deactivation of environmental contaminants, CAPP-generating parameters, the construction of reaction–discharge systems, operation costs, and possibilities for scaling-up such systems are important considerations. Furthermore, the achieved degradation efficiency can also be correlated with the CAPP gaseous atmosphere used and the type and content of generated RONS. Below, we show and discuss the three types of CAPP-based systems, dedicated to environmental applications, and having different plasma sources (Figure 5).

The first type of CAPP-based system used for the decomposition of environmental pollutants, such as antimicrobial agents, relies on planar dielectric barrier discharges (DBDs) [82,83,84,85,86,87,88,89,90,91]. The main parts of these systems are vessels to hold liquid or solid samples that are covered at the top and bottom with parallel plate electrodes and dielectric insulators. High voltages (HVs) with specific parameters, i.e., voltage amplitude (V_a_) within the range of 0.85–85 kV and discharge power (DP) of 0.86–1150 W supplied to these electrodes, result in the ionization of vast volumes of atmospheric air [82,83,84,85]. The most popular and relatively cost-effective working gas applied to treat environmental contaminants is air at a flowing rate of 1.0–4.0 L min^−1^ [82,83,84,85,87,92,93,94,95,96]. When CAPP-based systems are adopted to treat liquid samples with an increased efficacy, DBD reactors are constructed in such a way to enable the irradiation of thick liquid films of these samples or their whole volumes in large, closed containers. In this case, DBDs are sustained in propagating working gases that form a characteristic plasma plume [73,97]. Such systems, involved in the point treatment of solids or liquids, are experimental, laboratory-scale models possessing notable perspectives for rescaling [97].

A tempting alternative to the single use of DBD systems for the removal of organic compounds from studied solutions is employing both DBD and metallic ions in order to improve the degradation efficiency [98]. As an example, the utilization of Re(VII) ions as catalysts, together with DBD, might be provided [98]. It has been proven that Re nanoparticles (NPs) obtained and used directly in a CAPP-based system [98] enhance the degradation of chloramphenicol and furazolidone through their reduction to amino-derivatives. This outcome was possible because DBD, used as a CAPP, is a source of RONS, while ReNPs catalyze the whole reaction [98].

The second group of systems used for antibiotic degradation is based on the application of corona discharges (CDs) [86,99,100,101,102,103,104]. In these plasma-based systems, working metal electrodes (in the shape of needles, wires, or tubes) are connected to HV generators. By placing electrodes above the surface of liquid samples and applying high electrical potential between working and ground electrodes, CDs are ignited in contact with liquid samples [99,100,101,102,105]. In the most common systems, HVs of specific parameters, i.e., V_a_ in the range of 3–30 kV, DP enclosing in the range of 1.03–250 W, are applied to sustain CDs. To increase the effectiveness of their treatment, a number of metal electrodes are used to multiply the effect. CDs can be generated in bubbles by immersing metal electrodes in liquids and passing the working gas, usually ambient air at a flowing rate of 0.5–4.0 L min^−1^ [101,103]. An interesting approach for the removal of tetracycline from wastewater might be the utilization of pulsed CD, however, combined with natural soil particles [104]. In this case, the CDs operate under an air atmosphere, which is a quite similar to the procedures presented above; however, the addition of natural soil particles enhances the lifespan of the plasma channel. Associated with these findings, the removal efficiency of tetracycline increased [104].

The third type of systems designated for environmental protection is non-thermal or atmospheric pressure plasma jet (APPJ), and consists of a hollow electrode, through which a working gas is passed, connected to an HV supply. Providing a sufficient amount of energy to the hollow electrode, the fluxed working gas is ionized inside the hollow electrode and CAPP is sustained, propagating to open space in the form of a jet or a bullet [106]. APPJ systems can operate in either stationary or flow-through regimes, by placing these sources into containers with treated liquids, containing targeted contaminants [106].

The CAPP-based approach discussed within the three most commonly examined systems for organic pollutants possesses differences and similarities. The types of samples contaminated with organic pollutants that can be introduced into CAPP systems are liquids and solids. The utilization of DBD, CD, and APPJ discharge systems on these two types of samples has been described and discussed [107]. However, it should be mentioned that the penetration ability through solid samples would be especially limited for APPJ systems, and most likely for CD. On the other hand, the CD-based CAPP systems seem more practical in terms of construction. Taking into account the possible atmospheres for CAPP generation, each system can operate within the same gaseous atmosphere. However, for APPJ generation, the compression of atmospheric air is required, while DBD and CD systems can operate under atmospheric pressure. The construction complexity for DBD systems is relatively low, as in the basic scheme; the DBD reactor consists of two parallelly located dielectric electrodes that are driven with an HV source. However, the construction complexity can be increased, as solutions for falling films will be added for improved effectiveness. The construction complexity for CD-based systems seems low, as the system is composed of hollow or pin-type electrodes positioned above treated samples. Even by improving effectiveness by multiplying the electrodes, construction complexity remains low. Finally, the APPJ-based system construction remains relatively complex, as the electric and ground electrodes should be enclosed with a hermetically provided gas supply. Addressing the construction expenses of the discussed CAPP systems, DBD and CD are rather cost-effective, as in their basic form, the sole components include dielectric material or pin/hollow electrodes, respectively, wires, and HV supply. The contrary situation is for APPJ systems, where the expenses will be highly dependent on hermetic construction and hollow tube design. If the discharge atmosphere is reduced to the surrounding atmosphere/air, the operational costs for DBD and CD systems will be relatively low, including construction expenses. For the APPJ systems, the costs will be higher due to gas compression and construction expenses. Perspectives for scaling up the CAPP systems remain rather good for DBD and CD constructions, as they can be easily adapted for CAPP operation in batching or flowing regimes of polluted water, or on a conveyor belt for solids. It should be mentioned that, for APPJ systems, these perspectives are quite complicated. APPJ-based construction can be performed in flowing regimes of polluted water; however, the significantly enlarged size of the hollow tubes might be problematic, and the multiplication of plasma jets will consume more discharge gas. The described CAPP systems may present limitations. In the case of DBDs, following longer purification processes, the surfaces of plate electrodes can be covered within the evaporated pollutions, ions, and emulations present in treated water/soil, which results in the loss of their dielectric properties, disrupting CAPP discharge. In CD systems, the most troublesome issue can arise for pin electrodes, which during longer treatments consumed, etched, or covered with the evaporated constituents. The damaged pin electrodes shall be replaced or recovered to maintain discharge power. Following APPJ system operation, the most dramatic scenario includes problems with hollow electrodes that may collapse or clog due to pressure and electromagnetic field differences in discharge gap. Additionally, due to the CAPP ignition model in APPJ systems, the generation of flammable or explosive gases during the purification process may occur, posing potential risks for the operator. A comparison of these differences in CAPP systems is presented in Table 1.

There are also other examples of CAPP-based systems used for wastewater purification, applying either pulse-modulated radio frequency atmospheric pressure glow discharge (pm-rf-APGD) [108,109] or direct current atmospheric pressure glow discharge (dc-APGD) [109], and working in a continuous low mode. Such reaction–discharge systems have found application, for instance, in the decomposition of doxycycline [108] and endocrine-disrupting compounds (EDCs), including bisphenol A, bisphenol C, dexamethasone, benzophenone, and dapsone [109]. Due to the unique construction of these CAPP-based systems, developed technologies offer the efficient decomposition of targeted organic pollutants. One way to improve the degradation rate of organic compounds by pm-rf-APGD systems may involve the optimization of their operating parameters, such as flow rates and duty cycles, which can increase the efficiency of doxycycline decomposition [108], or the development of an APPJ-based plasma brush for the continuous removal of antibiotics from liquid solutions [67]. Recently, a catalytically enhanced approach was proposed for the degradation of organic pollutants. According to this concept, dc-APGD [98] is additionally supported by a nanocatalyst (ReOx nanoparticles formed in situ in treated media during the operation of a CAPP system), and enables the decomposition of furazolidone and chloramphenicol from studied solutions [110]. However, in this case, the procedure includes two steps, as follows. In the first step, the dc-APGD is applied for synthesizing ReNPs, and subsequently, the obtained nanostructures are mixed with the appropriate antimicrobial agent solutions for their degradation [110].

CAPP-based systems proposed thus far for the degradation of antibiotics are herein reviewed; their applications are summarized in Table 2. 

According to a number of scientific papers [82,83,84,85,86,87,88,89,90,91], CAPP-based systems built on DBDs are some of the most commonly selected in studies focused on the degradation of hazardous organics (Table 3, Figure 5). The main reason for such a common application of these systems is their simple construction and customizable structure. On the other hand, the operation of DBDs also might require expensive discharge gases, which raise expenses associated with the technological process. Furthermore, the use of noble gases limits the possibility of shifting plasma-based systems from one place to another, thus restraining their widespread implementation in real-life conditions. In juxtaposition to other CAPP-based systems, CDs as CAPP sources are rather user-friendly due to their simplicity of construction and highly effective output. However, energy consumption for these systems tends to be high, which might exclude them from industrial-scale applications [100]. For example, Ajo et al. [111] demonstrated the application of CD for aqueous oxalate oxidation [111]. In this case, the use of pulsed CD leads to an oxidation efficiency of around 36 g kW^−1^h^−1^ for the 100 W oxidation of aqueous oxalate [111]. Regarding CAPP jet systems, they seem to be less popular and bring fewer perspectives compared to the previously mentioned systems. Their construction and operational costs are high. As a remedy to the drawbacks presented above, the construction of CAPP systems could be adequately modified, while the operating parameters might be modulated, together with the up-scaling of devices, just to meet the demands of industrial-scale use [112].

Considering the organic pollutants related to agriculture, a whole variety of polycyclic aromatic hydrocarbons (PAHs), polychlorinated biphenyls (PCBs), and pesticides, among others, could not be omitted. Handling the elevated concentrations of these substances is, therefore, one of the most crucial challenges, bearing in mind the preservation of the natural environment [40]. Agricultural wastewaters contain these compounds which are highly resistant to deactivation, either by common wastewater treatment plants or natural environmental decomposition processes. As evidenced above, CAPP-based approaches are proven prospective technologies for the removal of a variety of pharmaceuticals, especially antibiotics. However, their applications might be wider, as a variety of CAPP-based techniques have already been recognized as effective towards the decomposition of organic compounds, named POPs. The recent advances in this field in the view of CAPP applications, both in soil and water solutions, are summarized in Table 3 [113,114,115,116,117,118,119,120,121,122,123,124,125,126,127,128].

In this view, DBD-based systems seem to be of particular utility among different CAPP-based approaches. Liu et al. developed an efficient method to degrade pyrene in soil using a tube array DBD plasma system. This study showed that DBD, operated under optimized working parameters, could degrade pyrene by up to 98.3%. Furthermore, the use of catalysts such as CeO_2_ and TiO_2_, along with the application of CAPP, can also enhance the degradation of pollutants in shorter times and improve efficiencies. DBD has also been used for the decomposition of toluene, 2,4,6-trinitroresorcinol, dichlorvos [113], malathion [113], endosulfan, trifluralin [116], phenol, 2,4-dichlorophenoxyacetic acid [118], 2,4-dichlorophenol [118,119], nitrobenzene and methylene blue [121,126,128], paracetamol, ceftriaxone, and caffeine [121], dimethyl phthalate [122], perfluorooctanoic acid [124], diatriazite [126], azo [127], and non-azo compounds [128]. In these cases, decomposition efficiencies within in the range of 70–100% [113,114,115,116,117,118,119,120,121,122,123,124,125,126,127,128] were achieved. It is worth mentioning that lower efficiencies were reached for compounds possessing additional nitro- and amino-groups (Table 3).

Considering the characteristics of the CAPP-based systems listed above, used either for antimicrobial agents or organic pollutant removal, the majority of systems work in stationary, non-flowing modes, limiting the volume of purified solutions. The improvement of the construction of CAPP-based systems might change their characteristics to a circulating one. However, the most effective are semi-continuous-flow CAPP-based systems or throughput systems. In these cases, the contaminated solutions subjected to CAPP treatment are continuously introduced to the systems, increasing the volume of purified solutions compared to stationary CAPP-based systems.

### 2.2. A Space for Improvement—Reactive Oxygen and Nitrogen Species

The mechanism of CAPP action is related to generation of a “cocktail” of RONS [65]. The type of produced RONS, their concentration, and lifespan depend on many factors, such as the type of working gas used for CAPP operation, the flow rate of this gas, the applied voltage and power, and the type of matrix treated by CAPP. The occurrence of specific reactive species, both long-lived and short-lived, is crucial for devising the mechanism of CAPP action [111,129].

Considering long-lived RONS, the most important are H_2_O_2_ and O_3_, while in terms of the short-lived molecules, ^1^O_2_ and ^•^OH are often listed [65]. Both types of species reveal oxidative potential to perform degradation and/or deactivation processes of active molecules, or further decomposition of the resulting by-products. For this reason, considerable attention is paid to the identification of RONS and the determination of their concentrations in view of the overall process of the CAPP deactivation of organic compounds (please refer to the Section 2 and Figure 3 for details).

Plasma–liquid interactions act as a gateway to understanding the degradation process of organic compounds that takes place above and in the liquid. These interactions not only influence the behaviour of RONS in relation to other species, but also between the defined RONS. Notably, the interplay between ROS and RNS is regarded as one of the most important aspects of plasma–liquid interactions. RNS contribute to plasma-driven processes to a lesser extent, including the degradation of biologically active compounds, leading to the lowering of the concentration of ROS by consuming them. Among different ROS, ^•^OH (short-lived), in addition to H_2_O_2_ and O_3_ (long-lived), were reported to be “more valuable” than RNS because they are more capable of efficiently performing plasma-associated procedures by facilitating additional oxidation processes [73,83,96,130].

ROS are classified as the main agents responsible for the degradation processes of water pollutants; therefore, the RNS presence leads to further recombination with free radicals and ions, forming oxygen-based intermediates with lower chemical activity. Furthermore, the RNS directly interact with each other, forming nitrous and nitric acids, organic alkyl peroxinitrites, and peroxinitrates in water environments. It is worth mentioning, that in natural groundwater, the contribution of metallic cations is present, which may further interact with nitrous and nitric acids, significantly inhibiting their activity. However, in a scenario demanding their additional suppression, several methods can be addressed. The most commonly applied technologies for RNS removal remain ion exchange, reverse osmosis, absorption, chemical and biological agents, and their combination [131]. To provide better efficiency, new agents are constantly being developed, including zeolite absorbents [132], chitosan absorbents [133], agricultural-waste-based absorbents [134], or microalgae [135].

In more detail, Nguyen et al. [87] determined that the most vital reactive species produced by CAPP during the degradation of antibiotics in hospital wastewater were ROS: in that case, O_3_, H_2_O_2_, and ^•^OH. They also revealed that the concentrations of long-lived ROS such as O_3_ and H_2_O_2_ and short-lived ROS such as ^•^OH were directly related to the CAPP treatment time [87]. Among various ROS, ^•^OH is considered as the most powerful nonselective oxidizing agent; however, its short lifetime in this case must be considered [87]. It was also reported that variation in pH influences the type and yield of ROS produced [87]. For instance, an increase in the pH of a solution results in an improved production of ROS, i.e., O_3_, H_2_O_2_, and ultimately ^•^OH, leading to enhancement in the degradation process of organic compounds

Here, significant research [82,83,84,136,137] that has contributed to elucidation of the roles of RONS in the process of antibiotic degradation is summarized. For example, using optical emission spectrometry (OES), Hatzisymeon et al. [82] detected several RONS, such as N_2_^+^, NO, ^•^OH, and atomic O, during the CAPP-driven procedure of antibiotic deactivation [82]. However, as stated above, most studies indicate the primary role of ROS in the degradation of pharmaceuticals. Within these, the degradation of enrofloxacin was mainly attributed to the presence of •OH and ^1^O_2_, which were listed as the most significant species involved in plasma–liquid interactions. The use of radical scavengers has revealed their roles, namely, 2,2,6,6-tetramethylpiperidine (TEMP) for ^1^O_2_ and D-mannitol for capturing ^•^OH. In this way, it was established that, by the inhibition of these ROS, the whole enrofloxacin degradation process was ceased [83]. In another study, H_2_O_2_, O_3_, and ^•^OH were designated as responsible for the degradation of OTC [93]. In addition, Cheng et al. [85] analyzed the contribution of O_3_ and H_2_O_2_ in the CAPP-mediated degradation of tetracycline chloride (TCH). In this case, it was reported that the generation of O_3_ and H_2_O_2_ leads to the formation of ^•^OH, which is further consumed by TCH due to its reactivity [85,138,139]. The latter case clearly shows that ^•^OH plays an important role in TCH degradation. However, the breakdown of AMX is attributed not only to the production of ^•^OH, but also to the generation of O_3_ and H_2_O_2_. In this context, the optimization of CAPP operating parameters in terms of the increased production of ROS could act favourably for the efficient degradation of pharmaceuticals. In this context, Nguyen et al. investigated the removal of antibiotics, such as CFX, OFX, cefuroxime (CX), and AMX, from hospital wastewater using CAPP. Optimal operating parameters were defined by monitoring changes in the degradation efficiency, probably due to pH, the applied voltage, the interelectrode distance, and the reaction time. These parameters were then considered in terms of the generated ROS, and linked with the antibiotic removal efficiency. Accordingly, in a similar study, Dzimitrowicz et al. [108] determined optimal CAPP operating parameters to increase the yield of doxycycline decomposition.

In addition to optimization of the working parameters of CAPP systems applied for the decomposition process, there is another approach that could be applied to facilitate the production of ROS. Based on several reports in the literature, the generation of ROS could be boosted by applying a catalyst into the degradation process, facilitated by CAPP operation. As such, the presence of catalysts (Fe^2+^–Mn^+^/AC) resulted in gradually decreasing the concentration of O_3_ (from 2.25 mg L^−1^ to 1.56 mg L^−1^) and H_2_O_2_ (from 205.8 μmol L^−1^ to 158.8 μmol L^−1^), observed after 15 min of CAPP treatment, and therefore stimulating the decomposition of TCH [85]. In effect, application of a catalyst improved the efficacy of the DBD system, as was apparent by the observed decomposition rate of TCH.

The idea of catalytically enhanced CAPP processes has been further examined, and is not only limited to the degradation of antibiotics. Markovic et al. examined the removal of ibuprofen in wastewater by employing three experimental methods: DBD (as a CAPP source) alone; DBD with Fe^2+^; and via the Fenton reaction, without plasma. Only 85% of ibuprofen was removed using DBD or Fenton reactions alone; however, by combining these processes, it was possible to achieve a 99% removal efficacy within the same time—in this case, 15 min [140]. In this study, Fe^2+^ stimulated the formation of ^•^OH and improved the degradation of ibuprofen [140]. The combined technology may be considered as safe, because ibuprofen degradation products did not exhibit toxic effects towards *Aliivibrio fischeri* bacteria [141], whereas the products of Fenton reactions did. The lack of toxicity of solutions treated by DBD and DBD/Fe^2+^ systems towards the above-mentioned marine bacteria was assigned to the higher yields of ^•^OH produced in these conditions [140,142]. Liang et al. [102] implemented a catalyst-mediated method to degrade trimethoprim (TMP) from water samples by using nanosecond pulsed gas–liquid discharge (NPG-LD) [102]. Application of this CAPP source activated persulfate S_2_O_8_^2−^ ions and significantly enhanced the efficacy of TMP degradation. According to OES analyses, ^•^OH and H_2_O_2_ also played a vital role in degradation of TMP. The concentration of ^•^OH was directly correlated with the TMP degradation rate [102], while the generated H_2_O_2_ contributed to the production of ^•^OH, as determined by other researchers [143,144,145] who applied magnetized carbon nanomaterial/NiFe_2_O_4_ together with a non-thermal APPJ during CAPP operation to decontaminate water polluted with CFX [146]. The degradation of CFX was conducted with the use of non-thermal plasma alone or in combination with activated carbon (walnut-based or charcoal), multiwalled carbon nanotubes (CNTs), NiFe_2_O_4_, and activated carbon charcoal–NiFe_2_O_4_. It was revealed that the application of a catalyst increases the life span of ROS, mainly ^•^OH, and thus contributes to a higher yield of CFX degradation.

### 2.3. Environmental Impact

#### 2.3.1. Biological Effects of CAPP Treatments

To assess biological effects, the potential impact of CAPP-treated solutions on living organisms, have been investigated by various research groups, studying either the antibacterial properties or toxicity of the treated liquids. For instance, Sarangapani et al. [147] revealed differences in the antimicrobial properties of antibiotic solutions exposed to a stationary reaction–discharge system under either 70 kV or 80 kV in contrast to untreated control solutions, as measured using disc-diffusion and microdilution tests. The outcomes of the disc-diffusion assay showed reductions in the antibacterial properties of CFX and ofloxacin (OFX) solutions against *Escherichia coli* ATCC 25,922 and *Bacillus atrophaeus* ATCC 9372 after treating them with CAPP. Regarding both antibiotics suspended in water and meat effluent matrices, the degradation of OFX turned out to be much more effective than that of CFX. Prolonged exposure of these solutions to CAPPs, from 15 min to 25 min, led to a more prominent reduction in the antibacterial properties of these antibiotics. In general, the exposure duration, the type of antibiotics, and the sample matrix influenced the efficacy of the CAPP treatment [115].

Interestingly, determination of the minimum inhibitory concentration (MIC) by conducting the microdilution assay gave contradictory results: CFX showed higher antimicrobial activity after the CAPP treatment than the corresponding control solution. In more detail, the impact of drugs was boosted fourfold in the case of *E. coli*, and twofold regarding *B. atrophaeus.* At the same time, the MIC of OFX treated by CAPP increased, which agreed with the outcomes of the disc-diffusion assay. This discrepancy in the results collected by various testing methodologies was likely associated with the complexity of the cascade processes following CAPP treatment [115].

Similar experiments were conducted by de Witte et al. [148] while studying ozonation, and by Paul et al. [149] when examining UVA photocatalysis. The above-mentioned disc-diffusion approach was also implemented by Terefinko et al., [67] establishing percentage deviations in antibacterial properties of solutions of doxycycline, OFX, ampicillin, chloramphenicol and mixtures of these antibiotics treated by high-throughput, continuous-flow, CAPP-based systems, i.e., the plasma pencil or the plasma brush [67]. It was proven that application of the unique plasma brush significantly reduced the antimicrobial properties of antibiotic solutions by 12.1–81.6%, as shown in the case of four different bacterial strains, i.e., *E. coli* ATCC 25,922, *B. subtilis* ATCC 23,857, *Serratia marcescens* ATCC 14,756, and *Enterobacter cloacae* ATCC 13,047. On the other hand, a continuous-flow reaction–discharge system based on pulse-modulated radio-frequency atmospheric pressure glow discharge (pm-rf-APGD) turned out to be even more efficient in impairing the biocidal activities of antibiotics than previously mentioned systems [108]. By utilizing a disc-diffusion assay, it was revealed that a doxycycline solution completely lost its antibacterial properties against *E. coli* ATCC 25,922, and notably reduced them by 37% and 29% in relation to *Staphylococcus haemolyticus* ATCC 29,970 or *S. aureus* ATCC 25,904. Similar outcomes were noted by Zhang et al., [150] who documented the loss of antimicrobial properties of a cefixime solution towards *E. coli* ATCC 25,922 after CAPP treatment inside bubbles with an enlarged gas–liquid interfacial area.

The CD treatment of diclofenac and verapamil hydrochloride also resulted in notable decreases in the antibacterial properties of these drugs, as revealed in a disc-diffusion assay on *E. coli*. [151] In addition, this test confirmed that verapamil is more toxic than diclofenac. After a 12 min (flow rate 166 mL min^−1^) exposure, diclofenac was completely removed, whereas the activity of verapamil was deactivated after the 21 min treatment. An attempt to decay compounds disrupting homeostasis of the endocrine system was undertaken by Krause et al. [152]. A CD system over a thin layer of water, in which the counter electrode was immersed, was used. In this study, an estrogen active contrast medium, iopromide, completely lost its stimulatory effects on the proliferation of MCF-7 cells after 15 min exposure to the CD, as revealed by an E-Screen Assay approach [152].

All these examples confirmed that CAPP-based technologies not only offer high yields of pharmaceutical degradation, but also lead to decay products of decreased or eliminated antimicrobial activity or toxicity. In this view, these approaches not only lead to decreases in the concentrations of common, pressing contaminants, but also seriously limit microbial exposure to antibiotic residuals, which may be one of the options in the inhibition of the spread of multidrug resistance in the environment.

#### 2.3.2. CAPP-Based Strategies as a Tool for Limiting the Spread of Multidrug Resistance

**Figure 6:**
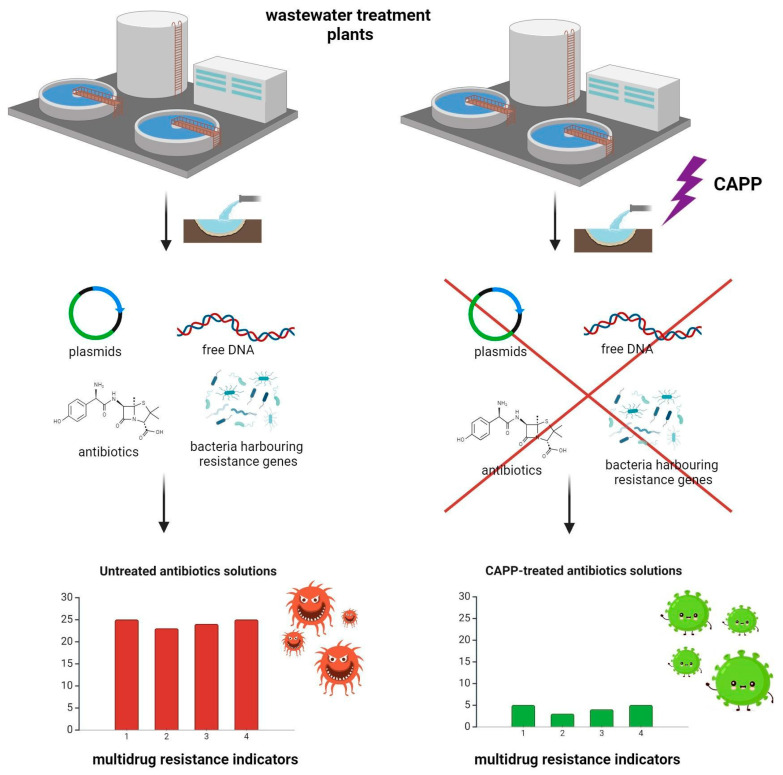
The contribution of the CAPP-based decontamination process to lowering multidrug resistance among bacterial pathogens.

The proposed CAPP-based methods enable the effective removal of pharmaceuticals and POPs from wastewater (Figure 6). Although the toxic characteristics of these contaminants are obvious, the occurrence of residual drugs in wastewater poses a serious, non-direct effect, namely, multidrug resistance among microorganisms. In this view, researchers thus far have investigated the applicability of cold plasmas to eradicate bacterial pathogens (including the drug-resistant strains) or eliminate DNA molecules containing drug resistance determinants, in addition to focusing on mechanisms explaining the transfer of antibiotic resistance genes or the destruction of CAPP-treated microbial cells [153]. Importantly, genetic determinants assuring antibiotic resistance tend to penetrate by horizontal gene transfer from harmless constituents of natural microbiota to pathogens dangerous to humans, such as *Enterococcus faecium*, *E. coli*, *S. aureus*, *Klebsiella pneumoniae*, *Acinetobacter baumannii*, or *P. aeruginosa* [154,155] (Figure 6). Moving back to antibiotic residuals and transformation products yielded by the plasma treatment, which are of the highest interest for this review, it is crucial to find an answer as to whether intermediate products of antibiotic degradation are capable of posing any selective pressure, leading to the spread of antibiotic resistance in bacterial populations. In this view, Sarangapani et al. [115] investigated whether sublethal doses of CAPP-treated CFX lead to bacterial adaptation to the presence of this drug in a similar manner as the untreated CFX [115]. During 24 h incubation in 37 °C, bacterial cells of *E. coli* ATCC 25,922 were exposed to CAPP-treated or untreated solutions of CFX present at concentrations changing from the twofold lower MIC value to one-eighth of this value, i.e., 0.04–0.6 μg L^−1^. Importantly, the antibiotic solutions treated with a stationary reaction–discharge system under variable voltage conditions for 25 min rendered *E. coli* ATCC 25,922 fivefold less resistant to CFX than the untreated solution of this chemotherapeutic agent.

Antibiotics released into the natural environment are responsible for a selective pressure that results in an increased abundance and broader distribution of resistance genes in bacteria found in soil or water [156,157]. For instance, fluoroquinolones persist in the environment [158,159,160]; their presence has been disclosed in municipal and surface waters in Switzerland [161,162], Sweden [163], and Vietnam [164]. Moreover, the presence of four antibiotics from the fluoroquinolone group, i.e., norfloxacin, CFX, lomefloxacin, and enrofloxacin, in tap water from 10 locations in Guangzhou province (China), ranged up to 82.7, 679.7, 179.0, and 8.3 ng L^−1^, respectively [165]. The impact of wastewaters contamination with antibiotics in addition to drug resistance genes in the natural environment is an important contemporary issue. Unfortunately, little research has been conducted in the context of CAPP contributions to limitations of the spread of antibiotic resistance among commonly occurring pathogenic microorganisms.

#### 2.3.3. Studies on the Putative Environmental Impact of CAPP-Treated Organic Solutions

Toxicity assays of CAPP-treated solutions should be conducted to assess their potential hazards to living organisms. In these tests, the EC_50_ value, a reference concentration, or an interaction threshold, determined by a biological dose–response model for given toxicity evaluation, are established [166]. The ecotoxicity of 2,4-dichlorophenoxyacetic acid was evaluated after treatment by CD using *Chloralle vulgaris*, a green eukaryotic microalga [167]. The toxicity of treated and untreated samples was confirmed by the percentage loss in microalgae cell viability. The authors revealed that the toxicity of carbofuran and its intermediates had dropped to zero after 10 min of pulsed corona discharge plasma treatment. Another study presented the results of an acute aquatic toxicity experiment involving two fish cell lines (PLHC-1 and RTG-2) and a crustacean model (*Daphnia magna*). Wastewater originating from the food processing industry was shown to be toxic to aquatic models; however, conducting the CAPP treatment with DBD reduced the observed toxic effects. Meat and dairy effluents were treated with DBD at 80 kV for 5 and 10 min. The concentration- and treatment-time-dependent cytotoxicity of the CAPP-treated effluents was observed for both cell lines, in which higher toxicity (i.e., >50%) was observed for concentrations above 10% of the CAPP-treated effluent. After 5 or 10 min from the CAPP treatment, toxicity of the 5% concentrated effluent dropped by 100% and 73%, as revealed with the *D. magna* model exposed for 24 h [118].

## 3. Conclusions, Perspectives, and Future Outlooks

CAPPs have emerged as promising technologies for the degradation of pollutants, such as antimicrobial agents and POPs, originating from various sectors, in particular from food production industry. Non-equilibrium conditions of CAPPs and the generation of RONS are responsible for the effectiveness of CAPPs, represented by DBD, CD, and APPJ systems, in deactivating organic pollutants without the need for additional reagents. Optimization of the operating parameters of such CAPP systems, in addition to their characteristics (non-flowing or continuous flowing), could certainly lead to increased degradation efficiencies of antibacterial agents and POSs by enhancing the production rate of RONS in these systems.

The production of reactive species, such as ^•^OH, O_3_, and H_2_O_2_, has been identified as a key factor in the potent degradation of organic pollutants. Optimization of CAPP-operating parameters may further improve the yield of these species, and as such contribute to the increased degradation efficiency of target pollutants. Additionally, as indicated in the review, the use of catalysts in degradation processes facilitated by CAPP systems is quite promising, since it results in changing the production rate of reactive species and further improving the degradation rates of target pollutants.

Prior studies have shown that CAPP treatments can lead to reductions in the antimicrobial activities and ecotoxicity of such treated solutions towards aquatic wildlife. These findings suggest that CAPP technologies not only remove target pollutants, but also may contribute to lowering the multidrug resistance among pathogenic microorganisms and limit the resultant healthcare risks.

Looking ahead, further research is warranted to explore and optimize the capabilities of CAPP-based technologies. This prognosis includes investigation of the long-term effects of CAPP treatments on microbiota and the whole complex natural ecosystems, as well as exploration of the scalability and cost-effectiveness of these technologies in industrial applications. Additionally, the development of innovative reactor designs, their integration with advanced catalysts, and the exploration of such combined treatments hold promise for enhancing the efficiency and sustainability of CAPP-based remediation systems. Following continued research and development, these approaches have a potentially significant role in addressing pollution challenges and ensuring the sustainability of food production processes.

## Figures and Tables

**Table 1:** Main aspects of applications of three CAPP-based systems for the degradation of antimicrobial agents and organic pollutants.

CAPP System	Samples Type	Atmosphere	Construction Complexity	Construction Expenses	Operational Expenses	Perspectives for Rescaling	Possible Limitation
DBD	Liquid and solid	Atmospheric air, nitrogen, oxygen, and argon	Relatively low	Relatively low	Relatively low	Good	Plates electrode surface oxidation, blocking their dielectric role.
CDs	Liquid and solid	Atmospheric air, argon, nitrogen, and oxygen	Low	Low	Relatively low	Good	Corrosion/decay/thermal melting of the discharge electrode.
APPJ	Liquid and solid	Compressed air, nitrogen, oxygen, argon, helium, and carbon dioxide	Relatively high	Relatively high	Relatively high	Disputed perspectives	Hollow electrode collapse/clogging. Explosion due to flammable/explosive gas generation.

**Table 2:** Recent studies presenting the degradation of antimicrobial agents using CAPP-based methods.

CAPP System	Operational Conditions *	Target Antibiotic	Removal Efficacy (%)	Degradation Products Ion Masses (m/z)	Reference
Nanosecond pulsed DBD reactor	CV = 26.6 kV, f = 200 Hz, A = ambient air, 1 L min^−1^, DP = 0.93 W, V = 8.5 mL, T = 20 min, stationary system	Enrofloxacin in water, 40 mg L^−1^	100	374, 356, 311, 243, 315, 362, 193, 293	[83]
Nanosecond pulsed DBD reactor	CV = 17.4 kV, f = 200 Hz, A = ambient air, 1.0 L mins^−1^, DP = 1.21 W, T = 3 min, stationary system	Ciprofloxacin in soil, 200 mg kg^−1^	99	412 (α-hydroperoxy amide and dihydroxy subset)	[84]
DBD reactor (with or without FeMn/activated carbon catalyst)	CV = 8.5 kV, A = ambient air, 3 L min^−1^, V = 500 mL, T = 15 min, stationary system	Tetracycline hydrochloride in wastewater, 50 mg L^−1^	73.4 (DBD alone), 98.8 (DBD/Fe-Mn/activated carbon)	461, 477, 448, 496, 416, 480, 405, 306, 274	[85]
DBD reactor	CV = 36.5 kV, SFR = 4 L min^−1^, T = 31 min, semi-flowing system	Amoxicillin in wastewater, 27.8–31.6 mg L^−1^	98.1	Several degradation pathways proposed (theoretical modelling)	[87]
DBD reactor	f = 600 Hz, A = air or oxygen, DP = 12.9 W, V = 100 mL, T = 40 min, circulating system	Amoxicillin, 100 mg L^−1^ sulfamethazole, 80 mg L^−1^ in water	100	NA	[92]
DBD reactor	DP = 8.9 W, f = 60 Hz, A = dry air, V = 1000 mL, T = 20 min, stationary system	Lincomycin, ciprofloxacin, enrofloxacin, chlortetracycline, oxytetracycline, sulfathiazole, sulfamethoxazole, sulfamethazine, trimethoprim, in wastewater, 5 mg L^−1^	100	NA	[93]
DBD reactor	CV = 10 kV, A = ambient air, 1 L min^−1^, stationary system	Oxytetracycline in wastewater, 100 mg L^−1^	93.6	476.4, 432.3, 388.3, 340.3, 288.3, 239.3	[94]
Pulsed DBD reactor with falling film	CV = 22 kV, f = 100 Hz, A = ambient air, 3.5 L min^−1^, SFR = 0.5 L min^−1^, T = 30 min, circulating system	Tetracycline in water, 50 mg L^−1^	92.3	416, 428, 461, 477, 384, 224	
DBD reactor	CV = 20 kV, f = 9.22 kHz, I = 1.05 A, V = 200 mL, SFR = 2 L min^−1^, A = ambient air, 3 L min^−1^, DP = 95.2 W, T = 20 min, circulating system	Tetracycline in water, 200 mg L^−1^	96.5	416.1, 418.1, 303.1, 365.1, 406.1	[96]
DBD reactor	DP = 90 W, f = 9.1 Hz, A = atmospheric air, V = 20 mL, T = 5 min, stationary system	Chloramphenicol, 30 mg L^−1^, furazolidone, 30 mg L^−1^, either in water or wastewater	Both 99.0	Amino-functionalized moieties of antibiotics	[110]
Pulsed CD reactor	SFR = 4.5 L min^−1^, DP = 60 W, T = 24 min, circulating system	Sulfamethizole in water, 50 mg L^−1^	100	OH-sulfamethizole, 3 OH-sulfamethizole, 4 OH-sulfamethizole, carboxy-sulfamethizole	[99]
CD above surface^1^, air bubbling CD^2^	CV = 3 kV, f = 5 kHz, I = 3 mA, A = ambient air, 1 L min^−1^, V = 50 mL, T_1_ = 90 min, T_2_ = 20 min, stationary system	Oxytetracycline hydrochloride, 50 mg L^−1^, doxycycline hyclate, 50 mg L^−1^, in water	70 (coxytetracycline^1^) 97 (doxycycline^1^) 100 (oxytetracycline^2^) 100 (doxycycline^2^)	NA	[101]
Pulsed CD reactor	CV = 22 kV, I = 180 A, SFR = 4.5 L min^−1^, circulating system	Amoxicillin, doxycycline, in water, 50 mg L^−1^	both 100	OH-amoxicillin, amoxicillin penicilloic acid OH-doxycycline and 2-OH-doxycycline	[100]
Nanosecond pulsed CD	CV = 30 kV, f = 150 Hz, A = argon or air, 200 mL min^−1^, DP = 1.03 W, V = 15 mL, T = 50 min, stationary system	Trimethoprim in water, 40 mg L^−1^	94.6	short-chain carboxylic acids, CO_2_, H_2_O, NH_4_^+^, NO_3_^−^	[102]
Air bubbling CD	CV = 8 kV, f = 8 kHz, A = ambient air, 0.5 L min^−1^, V = 40 mL, T = 50 min, stationary system	Amoxicillin in water, 1 mg L^−1^	99.9	CO, CO_2_, H_2_O, diketopiperazine 366, 349, 196, 147, 79, 115, 90, 60	[103]
Pulsed CD with natural soil particles	f = 75 Hz, A = air, 6.0 L min^−1^, V = 300 mL, T = 10 min, circulating system	Tetracycline in wastewater, 50 mg L^−1^	59.30 for black soil particles	444, 461, 416, 400, 274, 238, 209, 149	[104]
CD reactor	CV = 30 kV, A = ambient air, 4 L min^−1^, V = 600 mL, T = 15 min, stationary system	Ofloxacin, 41.2 mg L^−1^, ciprofloxacin, 1.0 mg L^−1^, cefuroxime, 0.3 mg L^−1^, amoxicillin, 23.6 mg L^−1^, in wastewater	72.1 99.6 99.2 75.8	NA	[86]
Argon APPJ	f = 670 Hz, DP = 32.6 W, A = argon, 2.5 L h^−1^, V = 30 mL, T = 24 min, stationary system	Ciprofloxacin in wastewater, 10 mg L^−1^	93.4	363, 307, 263, 261, 347, 288	[106]
pm-rf-APGD	f = 50 kHz, A = ambient air SFR = 2.8 mL min^−1^, continuous flow system	Doxycycline in water, 51.5 mg L^−1^	79.0	417.1649, 461.1541	[108]
pm-rf-APGD	f = 2300 Hz, duty cycle = 30%, A = ambient air, SFR = 3.0 mL min^−1^, continuous flow system	Bisphenol A, bisphenol S, dexamethasone, benzophenone, 2-nitrophenol, 17-alpha-ethinylestradiol, dapsone, in the 7-component mixture in water, 1 mg mL^−1^	58.8 28.2 28.5 74.5 61.7 71.5 92.8	NA	[109]
dc-APGD	CV = 1200 V, I = 30 mA, A = ambient air, SFR = 3.0 min^−1^, continuous flow system	Bisphenol A, bisphenol S, dexamethasone, benzophenone, 2-nitrophenol, 17-alpha-ethinylestradiol, dapsone, in the 7-component mixture in water, 1 mg mL^−1^	58.6 36.9 35.4 68.6 58.0 75.0 69.0	NA	[109]
APPJ plasma brush	f = 66 kHz, duty cycle = 52%, A = helium, 7.0 L min^−1^, SFR = 1.0 mL min^−1^, continuous flow system	Ofloxacin doxycycline ampicillin chloramphenicol in water, 10 mg mL^−1^	39.77 51.37 72.33 34.33	Detailed degradation pathways proposed for each antibiotics	[67]

* CV, current voltage; I, discharge current; DP, discharge power; f, frequency; A, atmosphere; V, treated volume; T, treatment time, SFR, solution flow rate.

**Table 3:** Recent studies presenting the degradation of various organic pollutants using CAPP-based methods.

CAPP System	Operational Conditions *	Pollutant Used	Removal Efficacy (%)	References
Tube array DBD reactor	CV = 28 kV, f = 9 kHz, A = nitrogen (80%) and oxygen (20%), 200 L min^−1^, T= 10 min, stationary system	Pyrene in soil, 100 mg kg^−1^	96.2	[113]
DBD reactor	A = ambient air, 3 L min^−1^, DP = 370 W, V = 500 mL, SFR = 450 mL min^−1^, T = 120 min, circulating system	2,4,6-Trinitroresorcinol in water, 135 mg L^−1^	100.00	[114]
DBD reactor	CV = 80 kV, f = 50 Hz, V= 20 mL, T = 8 min, stationary system	Dichlorvos, 850 µg L^−1^, Malathion, 1320 µg L^−1^, Endosulfan, 350 µg L^−1^, in water	78.98 69.62 57.71	[115]
Coaxial DBD plasma micro-discharges with high-voltage nanosecond pulses reactor	CV = 26.8 kV, f = 100 Hz, A = Air, 0.075 L min^−1^, DP = 1–2 MW, I = 75 A, T = 10 min, stationary system	Trifluralin in soil, 200 mg kg^−1^	99.5	[116]
Coaxial DBD reactor	CV = 15 kV, A = Air, 2.5 L min^−1^, V = 500 mL, T = 90 min, circulating system	Phenol, 2,4-Dichlorophenol (DCP), both in wastewater, 50 mg L^−1^,	56.17 89.55	[117]
DBD with falling film reactor	A = Argon, 1 L min^−1^, DP = 200 W, V = 500 mL, T = 15 min, circulating system	2,4-dichlorophenoxyacetic acid, 2,4-dichlorophenol, both in water, 100 mg L^−1^	100	[118]
DBD reactor	CV = 20 kV, A = Argon, 1 L min^−1^, V = 3 mL, T = 2 min, stationary system	2,4-dichlorophenol (2,4-DCP) in water, 400 mg L^−1^	98.16	[119]
DBD reactor	CV = 1.8 kV, A = Oxygen, 3 L min^−1^, DP = 0.538 W, SFR = 1.0 m^3^ h^−1^, T = 60 min, circulating system	Nitrobenzene in water, 20 mg L^−1^	75	[120]
DBD reactor with falling film	CV = 20 kV, f = 20 kHz, A = oxygen, 0.18 L min^−1,^ DP = 45 W, V = 100 mL, SFR = 90 mL min^−1^, T = 5–25 min, circulating system	Methylene blue, 20 mg L^−1^ Phenol, 50 mg L^−1^ Paracetamol, 25 mg L^−1^ Caffeine, 50 mg L^−1^, Ceftriaxone, 5 mg L^−1^, in wastewater	92 (T = 10 min) 100 (T = 20 min) 100 (T = 15 min) 100 (T = 20 min) 100 (T = 5 min)	[121]
Self-pulsing discharge (SPD)^1^ multipin corona discharge (MCD)^2^	CV = 30 kV, DP = 3 W, A = Air, 100 mL min^−1^, I = 12 mA, V = 50 mL, T = 30 min, stationary system	Dimethyl phthalate in contaminated water, 0.00002 mol L^−1^	91	[122]
Coaxial DBD reactor	DP = 40 W, A = CH_4_, 40 mL min^−1^, T = 2.86 s, gaseous system	Toluene in gas, 33 g Nm^−3^	85.9	[123]
Self-pulsing streamer discharge (SPD) reactor	CV = 30 kV, I = 12 mA, f = 100 Hz, DP = 2.89 W A = Argon, 100 mL min^−1^ V = 15 mL, T = 30 min, stationary system	Perfluorooctanoic acid (PFOA) in contaminated water, 41.4 mg L^−1^	84.0	[124]
Gas–liquid two-phase DBD reactor	CV= 17.6 kV, DP = 15 W, A = Air, 60 mL mins^−1^, T = 30 min, stationary system	Phenol in water, 1.06 mmol L^−1^	95.5	[125]
Pin-to-liquid discharge reactor	CV = 10 kV for MBD, 11 kV for DTZ; f = 1 kHz for MBD, 3 kHz for DTZ, A = oxygen; V = 7.5 mL, T = 11–20 min, stationary system	Methylene blue dye (MBD), 7 mg L^−1^, Diatrizoate (DTZ), 0.2 mg L^−1^, Both in water	84 (T = 11 min) 90 (T = 20 min)	[126]
Atmospheric-air-assisted GD reactor	CV = 6 kV, f = 100 Hz, A = atmospheric air, V = 20 mL, T = 30 min, stationary system	Orange G, Congo red, Crystal violet, Coomassie brilliant blue, all in water, 5 mg L^−1^,	99	[127]
Continuous-flow electrohydraulic plasma discharge (EHPD)	F = 60 Hz, DP = 300 W, A = Air, 3 L mins^−1^, V = 150 mL, SFR = 68 mL min^−1^, T = 10 min, circulating system	Methylene blue (MB) in water, 100 mg L^−1^	97.69	[128]

* CV, current voltage; I, discharge current; DP, discharge power; f, frequency; A, atmosphere; V, treated volume; T, treatment time; SFR, solution flow rate.

## List of Abbreviations

A	Atmosphere
AMR	Antimicrobial resistance
AMX	Amoxicillin
AOPs	Advanced oxidation processes
AOT	Advanced oxidation techniques
APGD	Atmospheric glow discharge
APPJ	Atmospheric pressure plasma jet
CAPPs	Cold atmospheric pressure plasmas
CD	Corona discharge
CFX	Ciprofloxacin
CNTs	Carbon nanotubes
CV	Current voltage
CX	Cefuroxime
DBD	Dielectric barrier discharge
DC	Direct current
DDT	Dichlorodiphenyltrichlorethane
D-man	D-mannitol
DP	Discharge power
EC_50_	Half-maximal effective concentration
EHPD	Electrohydraulic plasma discharge
ERM	Erythromycin
f	Frequency
Fe-Mn/AC	Iron–manganese/activated carbon
FOPs	Fenton oxidation processes
HV	High voltage
I	Discharge current
MDR	Multidrug resistance
MIC	Minimum inhibitory concentration
NPG-LD	Nanosecond pulsed gas–liquid discharge
NTP	Non-thermal plasma
OES	Optical emission spectroscopy
OFX	Ofloxacin
OTC	Oxytetracycline
PAHs	Polycyclic aromatic hydrocarbons
PCB	Polychlorinated biphenyls
PCDD	Polychlorinated dibenzo-p-dioxins
PCDF	Polychlorinated dibenzofurans
pm-rf	Pulse-modulated radio frequency
POPs	Persistent organic pollutants
RONS	Reactive Oxygen and Nitrogen Species
SAX	Sulfamethazole
SFR	Solution flow rate
T	Treatment time
TCH	Tetracycline hydrochloride
TEMP	2,2,6,6-tetramethylpiperidine
TMP	Trimethoprim
V	Treated volume
